# Incidence of IP and risk of malignant transformation in the Swedish population 1960–2010

**DOI:** 10.1007/s00405-016-4321-x

**Published:** 2016-10-18

**Authors:** Alexandra Elliot, Linda Marklund, Niclas Håkansson, Huan Song, Weimin Ye, Pär Stjärne, Lalle Hammarstedt-Nordenvall

**Affiliations:** 1 Department of Oto-Rhino-Laryngology, Head and Neck Surgery, Karolinska Institutet, Karolinska University Hospital, 171 76 Stockholm, Sweden; 2 Institute of Environmental Medicine, Karolinska Institutet, Stockholm, Sweden; 3 Institute of Medical Epidemiology and Biostatistics, Karolinska Institutet, Stockholm, Sweden

**Keywords:** Inverted papilloma, Squamous cell carcinoma, Neoplastic cell transformation, Incidence, Registries

## Abstract

The true incidence of inverted papilloma (IP) is not yet known. From hospital-based studies, its incidence has been estimated to approximately 0.5/100,000 person years. Earlier hospital case studies have shown that IP can undergo a malignant transformation in 1–53 %. The frequency of its malignant transformation on a population basis is unknown. To our knowledge, no standardised incidence ratio (SIR) has been reported for malignancies among IPs. This study aims to investigate these incidences on a population basis. Using the data from the Swedish Cancer Registry (SCR), we have identified patients with IP and patients with Squamous Cell Carcinoma (SCC) diagnosed between 1960 and 2010 in Sweden. Incidence of IP and incidence of SCC among patients with IP and SIR were analyzed. Eight hundred and fourteen patients with IP were identified. The incidence of IPs reported to the SCR increased from 1960 to 2010. In this cohort, SCC was overrepresented, as compared with the general population. The incidence of IP in the Swedish population seems to have increased.

## Introduction

Inverted papilloma (IP), often referred to as schneiderian papilloma, is a locally destructive benign tumour of the sino-nasal mucosa with a tendency for malignant transformation and a high propensity for recurrence [1–3]. It arises from the transitional epithelium, the Schneiderian membrane. Histologically, IP typically comprises both exophytic and endophytic components. The tumour invaginates into the underlying tissue, hence its name [4].

The true incidence of IP is not yet known, nor is its incidence pattern over time. From hospital-based studies, the incidence has been estimated to approximately 0.5/100,000 person years [1, 5, 6]. Most patients are diagnosed in their 60s with a male predominance of 2–10:1 [7, 8]. Earlier hospital case studies have shown that IP undergoes a malignant transformation in 1–53 % [9, 10]. The frequency of its malignant transformation on a population basis is unknown. To our knowledge, no standardised incidence ratio (SIR) has been reported for malignancies among IPs. The malignancies can occur synchronously, diagnosed in the same initial lesion, or metachronously, arising in the same area where the IP had previously been removed. In the literature, synchronous cancers are more frequent [11].

The aim of the present population-based cohort study was to establish the incidence of IP over time in the Swedish population as well as the risk of concomitant and later malignancies in the sino-nasal mucosa in patients with IP.

## Materials and methods

### Subjects

The Swedish Cancer Registry (SCR) covers the whole Swedish population, today approximately nine million people. All malignant tumours as well as some precancerous tumours, comprising IP, are reported to the SCR since 1958. The reliability of the SCR is high and the reporting to SCR is estimated to over 96 %, at least regarding true malignant neoplasms [12, 13]. Approximately, 99 % of the cases are morphologically verified [12]. The SCR reports cases using the ICD-7. Clinical diagnoses according to ICD-8 to ICD-10 have been transformed to ICD-7 in the register.

From the SCR, we included all tumours in the sino-nasal area, numbered 160.0, 160.2, and 160.7–160.9, according to the ICD-7, with a histological code for true papillomas. We chose to include patients reported to the SCR with IP since 1960, since the reporting to the register is considered reliable since 1960 [13]. Through the data from SCR, we were able to identify other information, such as gender distribution and age, at diagnosis (Table 1). In a similar way, we also retrieved data about those patients who were diagnosed with an SCC in the sino-nasal mucosa.

**Table 1:** Characteristics of patients with IP and SCC in Sweden 1960–2010

	1960’s	1970’s	1980’s	1990’s	2000’s	Total
IP						
*N* men	3	36	165	148	232	584
Mean age	40.7	57.6	52	54.9	56.3	54.7
*N* women	3	15	69	48	95	230
Mean age	47.7	54	55.9	52	59	56.1
*N* total	6	51	234	196	327	814
Mean age	44.2	56.5	53.1	54.2	57.1	55.1
Men/women	1	2.4	2.4	3.1	2.5	2.5
SCC						
Total			1	4	6	11
Mean age			66	69.5	61.2	64.6

### Statistics

Using the emigration and immigration registers from Statistics Sweden, we were able to identify patients lost to follow-up. Data concerning the size of the population and its age and sex distributions from year to year were also given by Statistics Sweden.

Because of the rarity of IP, we chose to report its incidence per decade. The calendar period was divided into decades beginning in 1960. Incidence rates were calculated by dividing the number of cases in each calendar period by the total average population in each age group in respective calendar period. The incidence rates were calculated for the whole population as well as sex specific. For comparison, the rates are also age-adjusted to the Swedish Standard Population of year 2000.

The risk of developing cancer among patients with IP was calculated as a proportion and a standardised incidence ratio (SIR). SIRs were calculated for SCC in patients diagnosed with IP by dividing the observed numbers of SCC in this group by the expected numbers of SCC based on person years at risk and population incidence.

Statistical analysis was done in SAS 9.2, STATA, and Excel.

The study was approved by the Ethical Committee at the Karolinska Institute, Stockholm, Sweden, according to the ethical permission 2012/49-31/2.

## Results

The mean age of diagnosis for IP was 55.1 (54.7 in male patients and 56.1 for women). No trend was seen over time (Table 1).

**Fig. 1:**
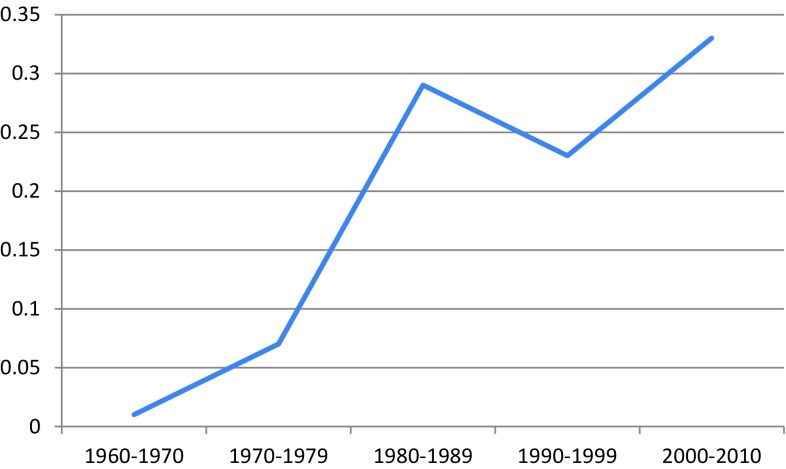
Incidence of inverted papilloma/100,000 py, age-adjusted Sweden, per decade

The male-to-female ratio in the 60s was 1:1, but only six cases were reported. The male-to-female ratio was rather constant over time from the 70s, 2.4–3.3:1 with no time-dependent trend. The incidence of IP increased over time with a substantial leap from the 60s when the incidence was 0.01/100,000 py (person years), to the 70s when the incidence was 0.07/100,000 py, and from the 70s to the 80s when the incidence was 0.29/100,000 py. After a smaller decrease during the 90s, *i* = 0.23/100,000 py, the incidence has again increased during the first decade of the twenty-first century to 0.33/100,000 py (Fig. 1). The same trend was seen in male and females.

Of all the persons who were diagnosed with IP, *n* = 814, 11 were diagnosed with SCC or SCC in situ which corresponds to a proportion of 1.35 % (Table 2). This represents an incidence of SCC over the whole time period of 111/100,000 py among patients with IP as compared with 0.37/100,000 py in the general population. Only in the male population did the IPs undergo a malignant transformation, which is a statistically significant (*p* < 0.05) difference compared with women. Among men with IP, the proportion developing SCC was 1.88 % with an incidence of 157.8/100,000 py as compared with 0.46/100,000 py in the general male population.

**Table 2:** Sex distribution of patients with IP and, among them, with SCC in Sweden 1960–2010

	*N* women	*N* men
IP	230	589
SCC	0	11

*N women* number of women diagnosed with IP and among them SCC, *N men* number of men diagnosed with IP and among them SCC

For the whole IP cohort, SIR was 142.76 with a *p* value <0.05, thereby also showing an over-representation of SCC among patients with IP. When analyzing the SIR stratified by duration, we found no evidence of reverse causality (Table 3).

**Table 3:** SIR of SCC in patients with IP stratified by duration of IP

*N*	Duration	Expected	Outcome	SIR	SIRL	SIRU	*p*
1	3–4 years	0.013621	1	73.42	1.86	409.06	0.0773
2	5–9 years	0.019131	3	156.81	32.34	458.28	0.0014
3	More than 10 years	0.037111	4	107.78	29.37	275.97	0.0003

*N* number of cases with IP who develop SCC, *duration* time between diagnosis of IP and of SCC, *expected* expected cases of SCC, *outcome* accumulated number of cases, *SIR* standardised incidence ratio, *SIRL* SIR lower, *SIRU* SIR upper, *p p* value

We did not find that patients with IP had a higher risk of developing sino-nasal malignancies of other histological types than SCC compared with the general population (data not shown).

## Discussion

In this population-based study, we found that during the study period, the age standardised incidence of IP increased by approximately 400 %, from 0.01 to 0.33/100,000 py.

The SIR of 142.76 showed a large over-representation of SCC among patients with IP (*p* < 0.005). Interestingly, the malignant transformation only occurred in men, representing a proportion of 1.88 % among men with IP. This represents a 300-fold increased risk as compared with the general male population.

In our study, two persons (18 %) with IP had synchronous SCC and nine (82 %) had metachronous SCC. Although our study was population based, our results regarding the incidence of IP are well in line with hospital data presented in Denmark [1, 14] and in the European position paper on the endoscopic management of tumours of the nose, paranasal sinuses, and skull base conducted by Lund et al. [15].

There are, however, some differences, where we found an age standardised incidence of 0.29 in the 80’s, whereas in Denmark, the incidence was 0.52 during approximately the same time period. This could be due to hazard but may also show the advantage of a higher coverage in a population-based study, or less accurate histopathological diagnosis.

In contrast to malignant sino-nasal tumours (malignant melanoma excluded) which have decreased over the decades [16], we found an increase of IP over time: In the 60’s, the incidence was very low (0.008), but from the 70’s, there has been a fivefold increase (0.006–0.033), an increase which has not been shown earlier. This may be a true increase. However, this could very possibly be due to underreporting during the earlier part of the study period and the possibility that over time more sino-nasal biopsies were sent for pathological assessment and diagnosis. The accuracy in the Swedish Cancer registry has been investigated for common, malignant tumours. IP is considered a precancerous lesion and the reporting rate may, therefore, be lower than for true malignant lesions, especially in the beginning of the study period. The increase is possibly also related to the detecting of IP due to a higher awareness of IP among ENT physicians and in their use of endoscopic sinus surgery. The preciseness of the histopathological evaluation has improved and may have lead to an increased reporting to the cancer register. The increase may also be correlated to a, until now, not fully identified environmental factor.

Earlier studies have reported diverging results concerning the risk of malignant transformation of 1–53 %, most of which are considerably higher than the 1.35 % that we found in our population-based study [8–10, 17, 18]. This discrepancy may be due to that earlier hospital-based studies from referral centres have dealt with patients with larger or recurrent IP tumours and that earlier studies, not based on a register with complete coverage of the population, (such as the SCR), have not included all IPs. It may also be due to differences in studied populations. Another plausible cause for the higher risk of malignant transformation in the previous studies is the likely underreporting of well-differentiated SCC “ab initio”. However, percentage does not allow comparison between studies on different populations, since difference in age or gender distribution is not known. Hopefully, future studies will be conducted presenting SIRs in other regions allowing a better comparison.

Our finding that only men with IP developed SCC has not been described earlier. To verify if this discrepancy between genders is true, larger cohort studies are needed. When considering the risk of SCC in the whole IP cohort, we should have found approximately five cases of SCC in the female population. In the review of the English literature conducted by Nudell et al. [10], 11 out of 59 cases of SCC ex IP occurred in women.

In the literature, synchronous SCC has previously been reported to be more common than metachronous malignant transformation [19]. In a meta-analysis including more than 2000 cases from 2007, Mirza and co-workers found a risk of 7.1 % of synchronous carcinomas compared with a risk of 3.6 % of metachronous malignancies [11]. Interestingly in our study, metachronous cancers were more than four times as common as the synchronous ones. A similar proportion was seen in only 4 of the 64 studies in the meta-analysis. Differing rates found in these earlier studies may be exaggerated due to referral bias to tertiary centres. Again, the difference in findings could be due to underreporting by the pathologist of IP when SCC is found in the same specimen. This needs to be investigated further.

## Conclusion

In this first population-based study, the incidence of IP has increased from 0.01 to 0.33/100,000 py in Sweden from 1960 to 2010. The risk of SCC malignancies in the IP cohort is lower than in the previous studies although significantly higher than in the general population. Interestingly, we only found SCC among male patients with IP where the risk of malignant transformation represented a 300-fold increased risk compared with the general male population.
